# A Case Series of Bacillary Layer Detachment (BALAD) in Neovascular Age-Related Macular Degeneration (nAMD): A Novel Optical Coherence Tomography (OCT) Biomarker

**DOI:** 10.7759/cureus.96819

**Published:** 2025-11-14

**Authors:** Konstantinos Flindris, Konstantina Gorgoli, Ioannis Koumpoulis

**Affiliations:** 1 Ophthalmology, General Hospital of Ioannina “G. Hatzikosta”, Ioannina, GRC; 2 Ophthalmology, Iris and Retina Private Practice, Ioannina, GRC; 3 Ophthalmology, General Hospital of Ioannina "G. Hatzikosta", Ioannina, GRC

**Keywords:** bacillary layer detachment (balad), case series, neovascular age-related macular degeneration (namd), oct angiography, oct biomarker, optical coherence tomography (oct), rare case

## Abstract

Bacillary layer detachment (BALAD) is a newly recognized optical coherence tomography (OCT) finding, defined as a split at the photoreceptor inner segment (myoid zone) resulting in a dome-shaped intraretinal fluid cavity. BALAD has been reported in various chorioretinal diseases and may serve as a novel structural OCT biomarker of severe exudation. We present a case series of BALAD in neovascular age-related macular degeneration (nAMD) to characterize its multimodal imaging features and discuss its diagnostic and prognostic significance. Three patients with active nAMD exhibited BALAD on spectral-domain OCT. In each instance, OCT cross-sections showed a sharply demarcated hyporeflective intraretinal cavity beneath an intact external limiting membrane (ELM) and above the ellipsoid zone (EZ). OCT angiography (OCTA) revealed an underlying high-flow choroidal neovascular network corresponding to the BALAD in all cases. All three patients initially had significant visual impairment. Two patients were treated with serial intravitreal anti-vascular endothelial growth factor (VEGF) injections, resulting in rapid resolution of the BALAD cavity, reabsorption of subretinal and intraretinal fluid, and marked improvement in best-corrected visual acuity (e.g., from 20/400 to 20/40). The third patient, who declined treatment, developed persistent BALAD with progressive subfoveal fibrosis and no visual recovery. BALAD in nAMD indicates an aggressive disease phenotype with intense outer retinal fluid accumulation and photoreceptor layer schisis. Prompt anti-VEGF therapy was associated with anatomical recovery and vision improvement in the treated cases. Recognizing BALAD on OCT and OCTA is clinically important, as it differentiates intraretinal from subretinal pathology and underscores the need for early, aggressive treatment to mitigate irreversible retinal damage in nAMD.

## Introduction

Bacillary layer detachment (BALAD) refers to a distinctive split within the photoreceptor layer, specifically at the inner segment myoid, producing an intraretinal fluid-filled cavity [1]. On optical coherence tomography (OCT), BALAD appears as a sharply demarcated hyporeflective pocket immediately below an intact external limiting membrane (ELM). Its anterior wall is formed by the remaining inner segment myoid, while the ellipsoid zone (EZ) and attached outer segments form the posterior floor. In practical terms, this means that BALAD represents a “fracture” through the photoreceptor inner segment: proximal photoreceptor cytoplasm with endoplasmic reticulum and other organelles is sequestered in the split, whereas the mitochondria-rich EZ and outer segment typically remain adherent to the retinal pigment epithelium (RPE) [2].

The term “bacillary layer detachment” (BALAD) was formally introduced to describe the inner segment split seen on OCT in macular retinochoroiditis due to toxoplasmosis [3]. Advanced OCT imaging has been critical for detecting BALAD. High-resolution spectral-domain OCT, first, enabled visualization of outer-retinal schisis, and even deeper-penetrating swept-source OCT has been used to capture BALAD. Clinically, identifying BALAD requires careful review of the outer retina on cross-sectional scans [4]. A key diagnostic clue is that the ELM remains intact over the cavity and the EZ forms its floor, a configuration not seen in a full-thickness neurosensory detachment. In practice, any unexpected cystoid space at the photoreceptor level should raise suspicion of BALAD [5].

BALAD has been reported so far in a broad spectrum of chorioretinal diseases. These mostly include neovascular age-related macular degeneration (nAMD), infectious and non-infectious posterior uveitis (e.g., Vogt-Koyanagi-Harada disease, ocular toxoplasmosis), pachychoroid spectrum disorders (including central serous chorioretinopathy), rhegmatogenous retinal detachment, blunt ocular trauma, and even neoplastic or paraneoplastic retinal diseases [6]. Across treatment-naïve nAMD cohorts, BALAD incidence ranges from roughly 4% to 7%, and in almost all cases, BALAD resolved following intravitreal anti-VEGF therapy over 4-48 weeks [7].

Herein, we present a case series of three patients with nAMD, all of whom demonstrated BALAD on OCT during the exudative phase of the disease. Through detailed multimodal imaging and clinical follow-up, we characterize the appearance and evolution of the BALAD in each case, correlate it with clinical findings, and assess visual outcomes. We further discuss the proposed pathophysiologic mechanisms underlying BALAD, its utility in diagnosis and prognosis of macular disease, and how this OCT sign can help distinguish intraretinal pathology across diverse etiologies. This series underscores BALAD as an emerging OCT-based novel structural biomarker and highlights its relevance in retinal imaging and patient care.

## Case presentation

This study is a retrospective observational case series of three patients who developed BALAD on OCT in the context of nAMD. The cases were selected from patients presenting to the Ophthalmology Department of General Hospital of Ioannina “G. Hatzikosta” and to the Iris & Retina Ophthalmology Center (Ioannina, Greece) during the past five years. All patients underwent detailed ophthalmic examination, including best-corrected visual acuity (BCVA) measurement, tonometry, dilated fundus examination, and multimodal imaging. Imaging modalities included color fundus photography, spectral-domain OCT, structural en face OCT of the macula, and OCT angiography (OCTA).

OCT and OCTA imaging were performed using Avanti widefield and AngioVue (Optovue Inc., Fremont, CA, USA), obtaining high-density macular scans through the fovea and areas of interest. Particular attention was given to identifying hyporeflective changes in the photoreceptor layer and outer retina. BALAD was defined as a dome-shaped, intraretinal cystic space bounded anteriorly by the ELM and posteriorly by the EZ. Clinical data, including the onset of the disease, treatments (if any), and follow-up findings, were collected from the medical records.

All patients gave fully informed consent for the use of their clinical data and images. Given the descriptive and retrospective nature of this case series, formal Institutional Review Board approval was not required or was exempted, and the study adhered to the tenets of the Declaration of Helsinki. Each case is detailed below in accordance with CARE case report guidelines, with identifying information omitted to maintain patient confidentiality.

Case 1

**Figure 1:**
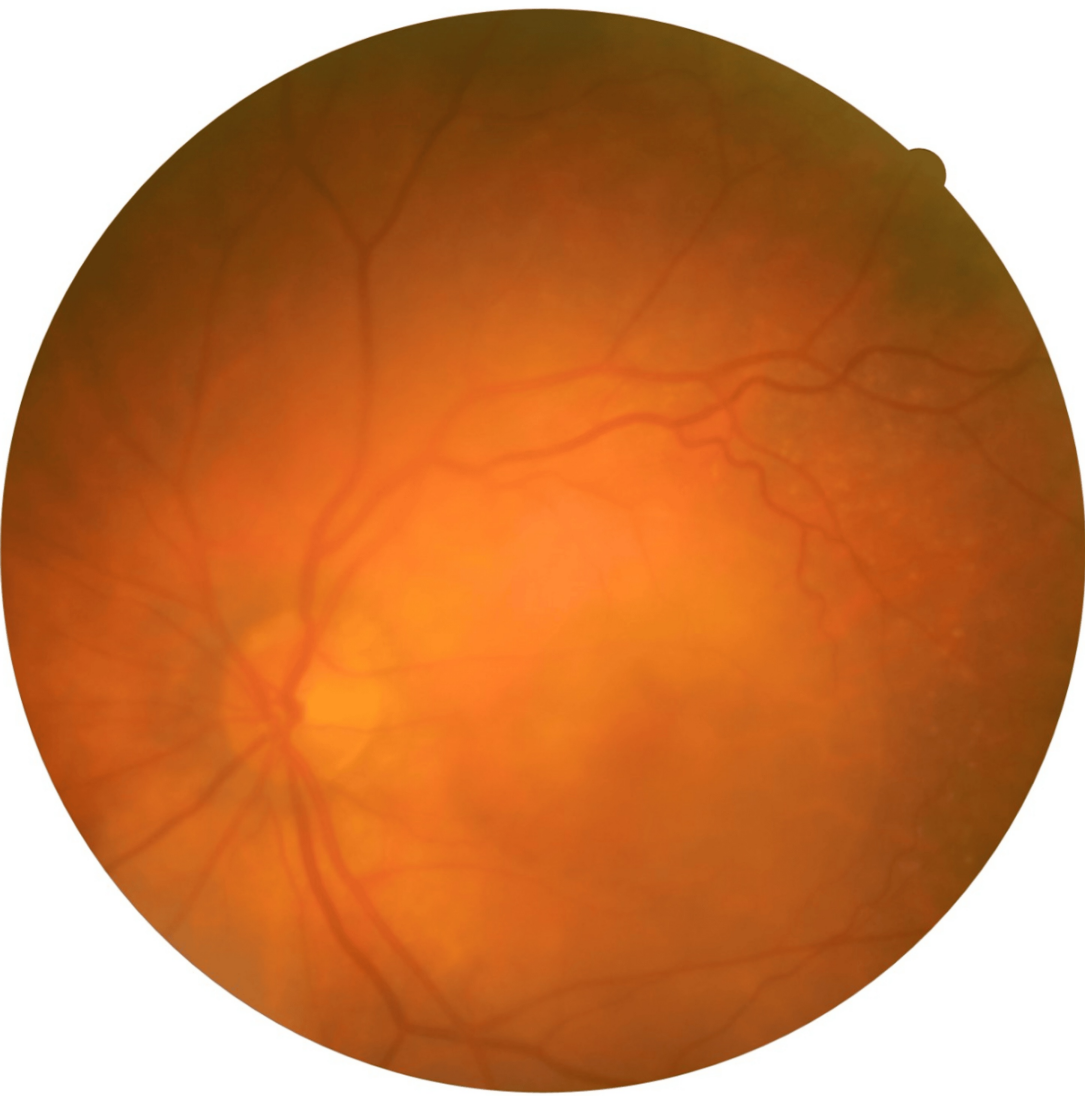
Color fundus photography of the left eye at presentation. A yellowish, well-circumscribed elevated lesion with light hemorrhage can be documented, highly suspicious of neovascular age-related macular degeneration (nAMD).

An 84-year-old woman on antihypertensive and anticoagulant therapy (apixaban 5 mg twice a day) presented with bilateral profound vision loss over the last months. The patient had a history of nAMD and received eight prior intravitreal anti-VEGF injections (aflibercept 2 mg) in the right eye, with the last injection performed about two years ago. At presentation, BCVA was counting fingers at 20 cm in both eyes. IOP was 15 mmHg bilaterally. Anterior segment examination revealed significant nuclear (3+) and posterior subcapsular cataracts in both eyes. Dilated fundus examination of the right eye showed a large submacular disciform scar with no active leakage on OCT (central retinal thickness (CRT) = 196 μm). By contrast, the left macula had a yellowish, well-circumscribed, elevated lesion with coexistent light hemorrhage (Figure 1), consistent with a neovascular AMD.

**Figure 2:**
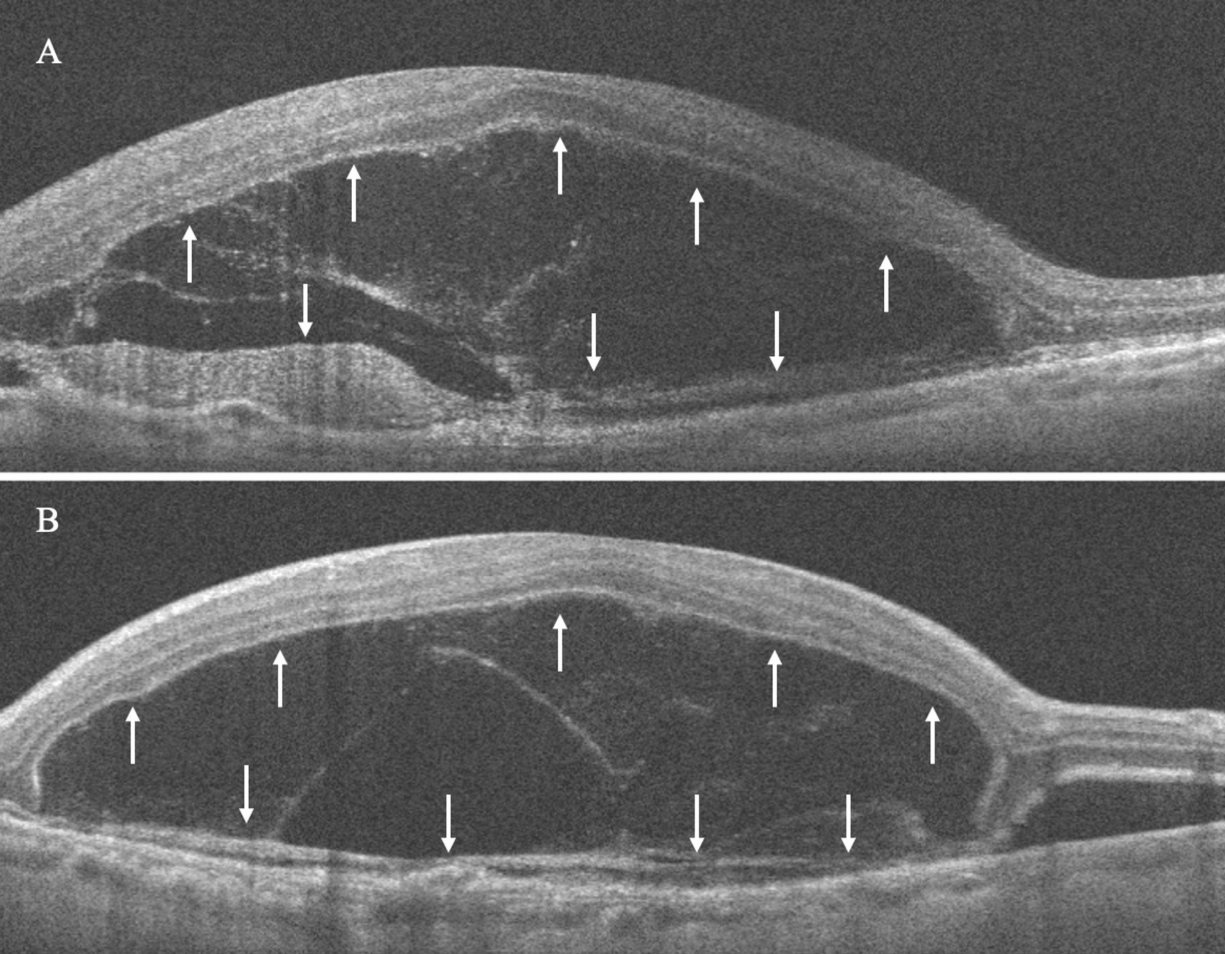
Spectral-domain optical coherence tomography (OCT) of the left eye at presentation (A horizontal scan and B vertical scan), revealing a large foveal elevation with splitting at the ellipsoid/myoid photoreceptor junction. The outer segments of the photoreceptors are attached. The hyporeflective cavity corresponds to a BALAD (arrows) while the hyperreflective bands within the lesion are suggestive of blood or fibrinous exudation.

**Figure 3:**
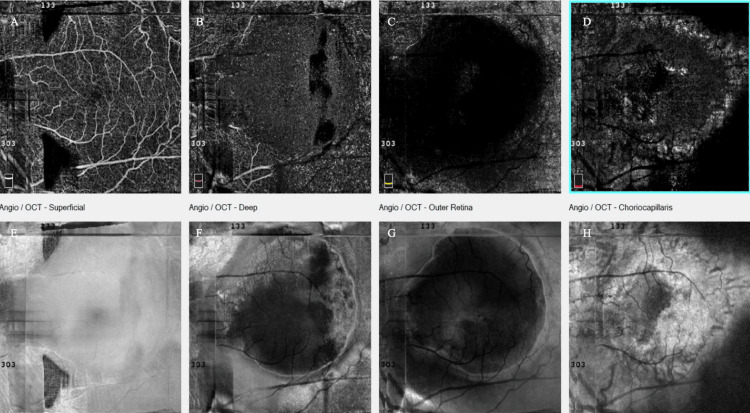
Optical coherence tomography angiography (OCTA) of the left eye at presentation, showing preservation of the superficial retinal vasculature (A), a broad round central flow void at the deep capillary plexus with artifact bands, corresponding to the area of the bacillary layer detachment (BALAD) (B), along with central loss of signal at the avascular layer masking the choroidal neovascularization (CNV) (C), central flow deficit with a mottled hyper/hypo-signal ring at the margins at the choriocapillaris layer, a pattern consistent with shadowing from overlying blood/fibrin and PED and not necessarily true choriocapillaris nonperfusion (D). Structural en face OCT (E-H) presented a large, well-circumscribed dome-shaped macular elevation with a hyperreflective rim and central hyporeflective core, matching the area of OCTA signal loss.

Spectral-domain OCT in the left eye revealed the presence of a BALAD, a large foveal elevation (CRT = 929 μm) with splitting at the ellipsoid/myoid photoreceptor junction and attached outer segments, creating a hyporeflective cavity. Moreover, hyperreflective bands were visible inside the cavity, suggestive of blood/fibrin within the BALAD (Figure 2). Associated findings included adjacent SRF and a shallow fibrovascular pigment epithelial detachment (PED) underlying the lesion. OCTA demonstrated preservation of the superficial retinal vasculature, whereas a broad, round central flow void at the deep capillary plexus with artifact bands, corresponding to the area of the BALAD, was revealed. The large circular area with complete loss of signal at the level of the outer retina was masking any underlying choroidal neovascularization (CNV) and probably corresponds to the BALAD margins. At the choriocapillaris layer, there was a central flow deficit with a mottled hyper/hypo-signal ring at the margins, a pattern consistent with shadowing from overlying blood/fibrin and PED, not necessarily true choriocapillaris nonperfusion. Furthermore, structural en face OCT presented a large, well-circumscribed dome-shaped macular elevation with a hyperreflective rim and central hyporeflective core, matching the area of OCTA signal loss (Figure 3). Thus, these OCT findings are characteristic of active nAMD accompanied by a BALAD.

**Figure 4:**
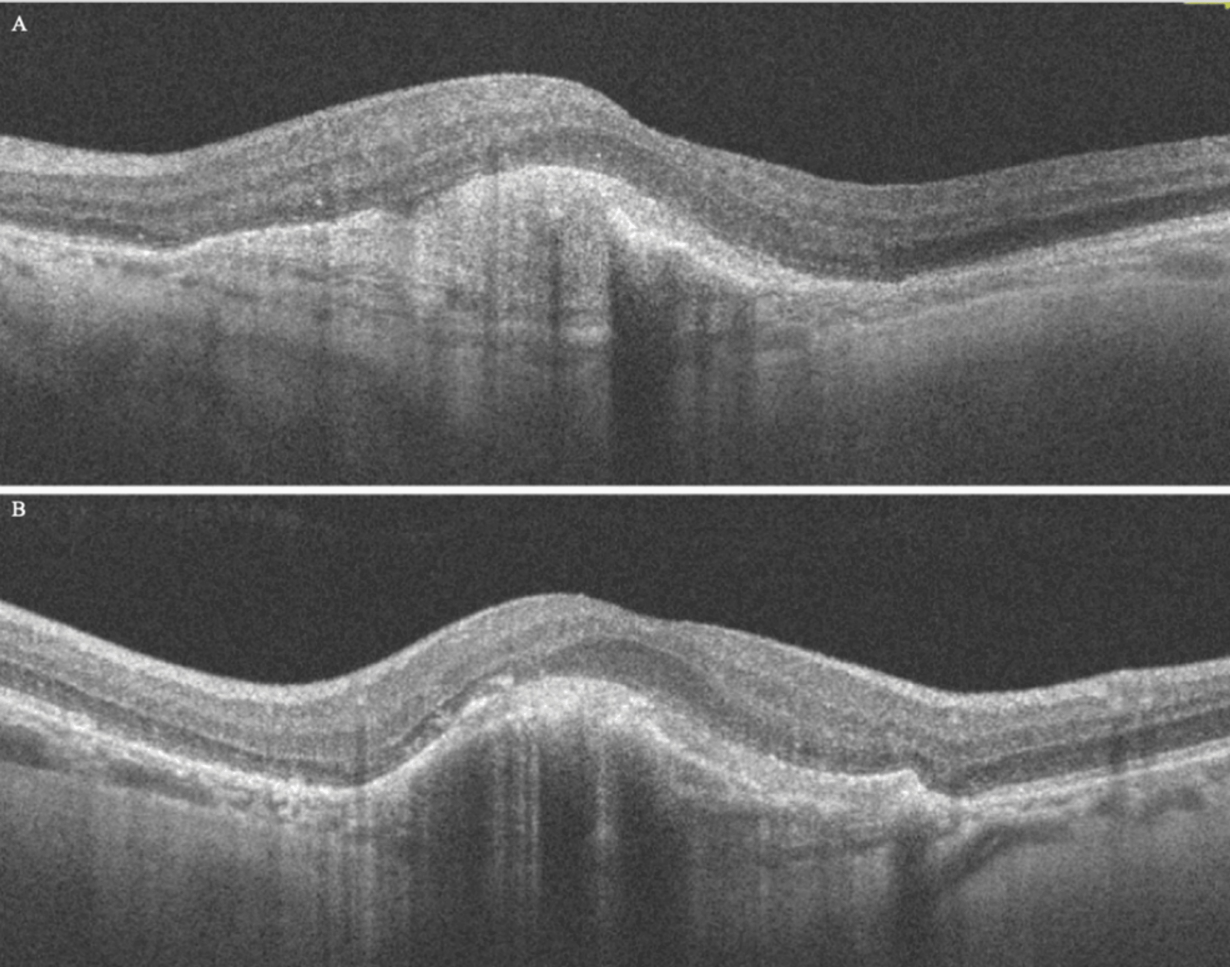
Spectral-domain optical coherence tomography (OCT) of the left eye at the 12-month follow-up (A horizontal scan and B vertical scan), showing marked reduction of retinal thickness, near-complete collapse of the bacillary layer detachment (BALAD), and only a residual foveal elevation due to fibrotic scar.

The patient was treated with six serial intravitreal anti-VEGF injections (aflibercept 2.0 mg) in the left eye, on a treat-and-extend regimen, while the cataract was removed via phacoemulsification. At the 12-month follow-up, BCVA in the left eye had improved to 20/50. OCT showed marked reduction of retinal thickness (CRT = 310 μm) and near-complete collapse of the BALAD; only a residual foveal elevation remained due to fibrotic scar (Figure 4). Overall, these findings illustrate a classic BALAD case in nAMD, as intense exudation and hemorrhage caused photoreceptor splitting, which then regressed with intravitreal anti-VEGF injections.

Case 2

**Figure 5:**
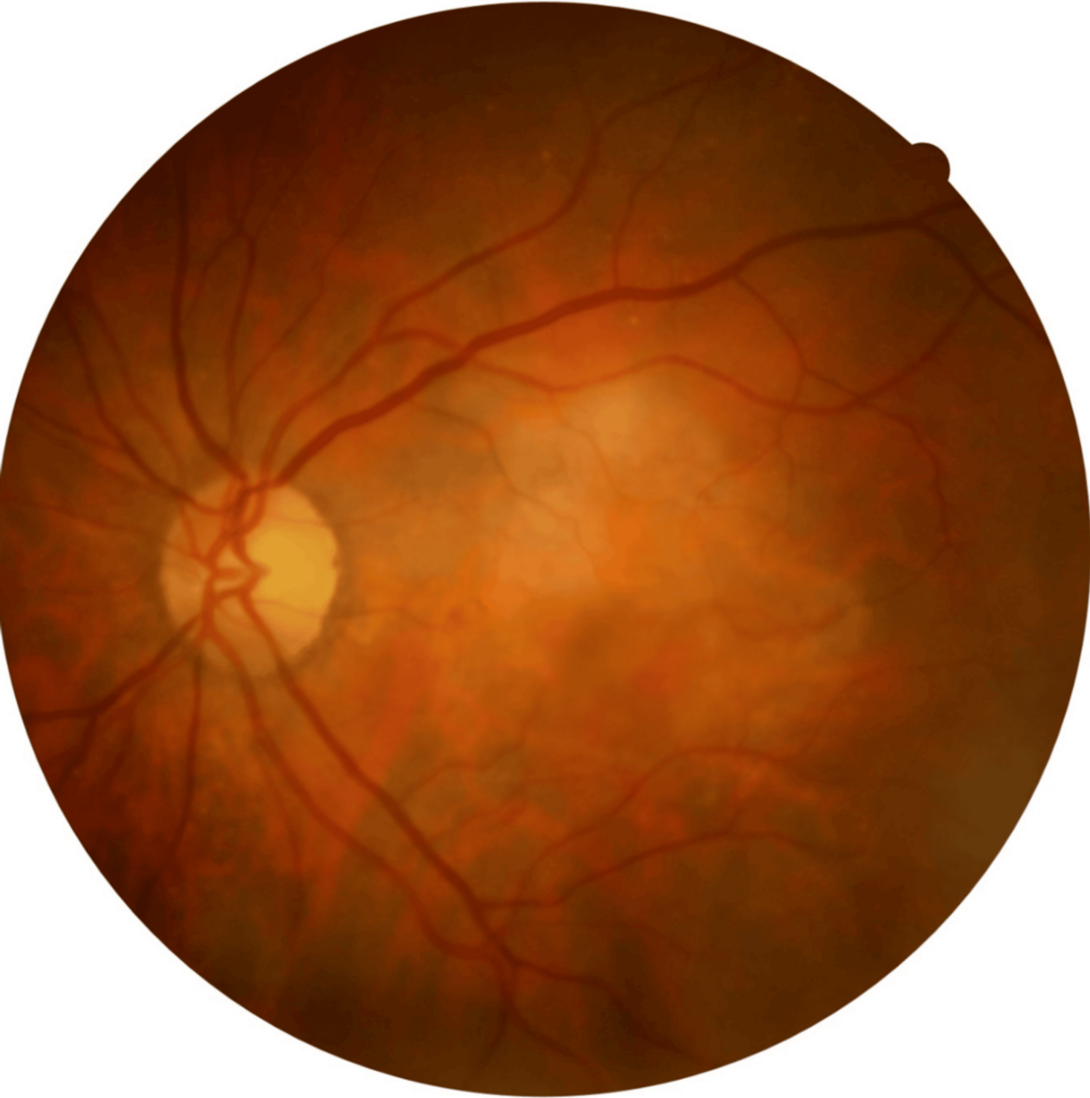
Color fundus photography of the left eye at presentation, exhibiting a round, yellow-gray elevation surrounded by a subtle hypopigmented ring with minor hemorrhage and exudation, corresponding to neovascular age-related macular degeneration (nAMD).

**Figure 6:**
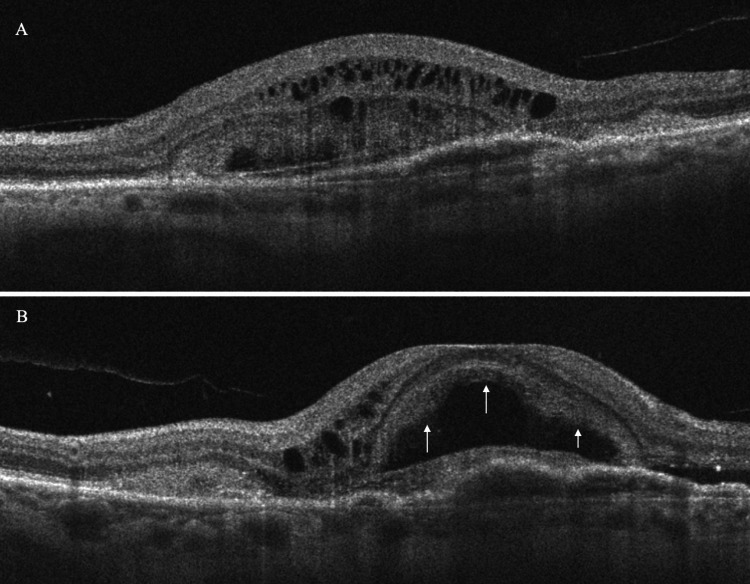
Spectral-domain optical coherence tomography (OCT) of the left eye at presentation (A horizontal scan and B vertical scan). Foveal photoreceptor splitting at the inner segment myoid, forming a dome-shaped intraretinal cavity with smooth, sharply demarcated walls, located between an intact external limiting membrane (ELM) and the ellipsoid zone (EZ) (bacillary layer detachment (BALAD) - arrows). Irregular pigment epithelial detachment (PED), significant fibrosis, presence of intraretinal fluid (IRF) and subretinal fluid (SRF).

**Figure 7:**
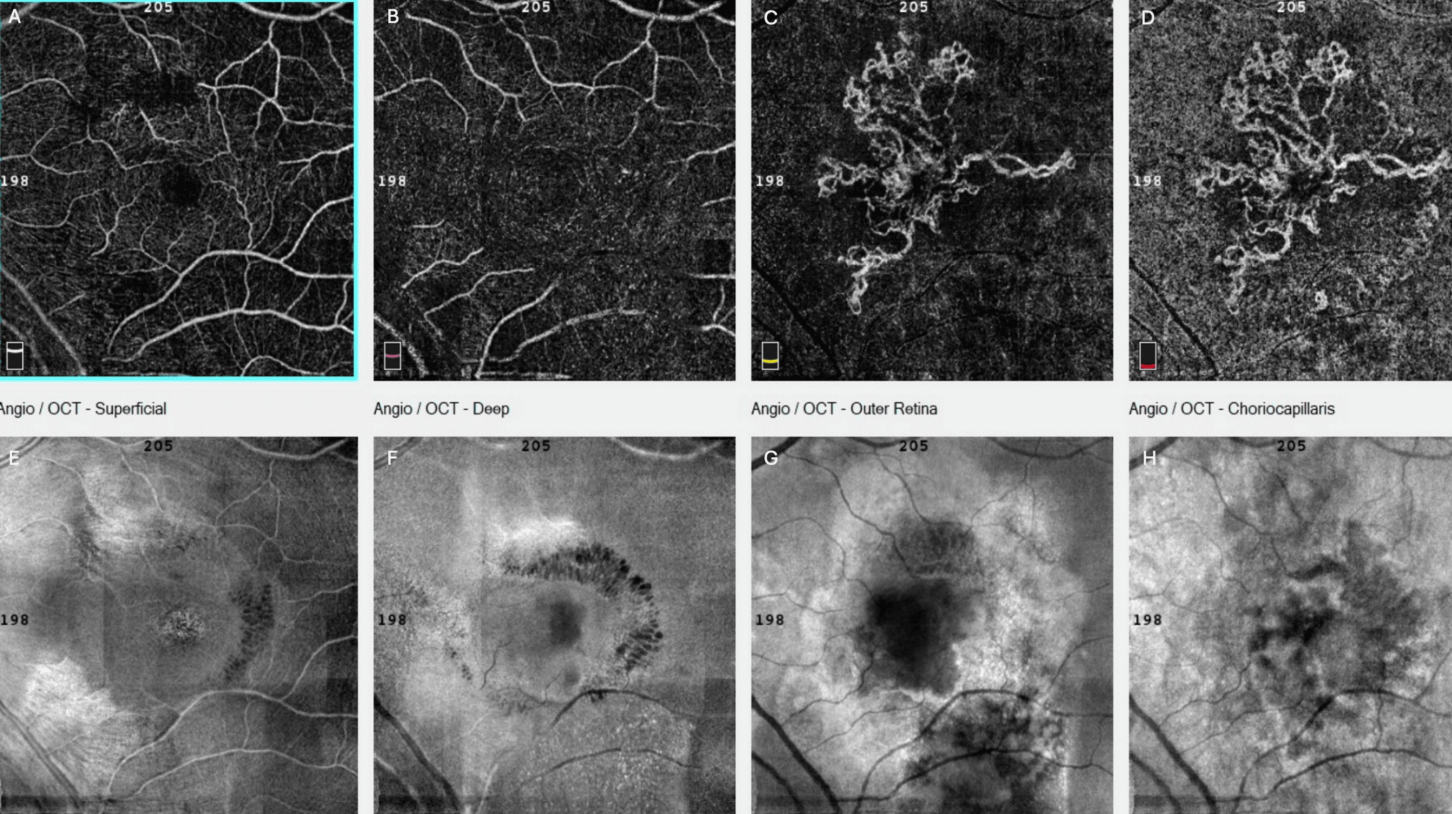
Optical coherence tomography angiography (OCTA) (A-D) of the left eye at presentation, displaying a well-defined, high-flow lacy-wheel/sea-fan choroidal neovascularization (CNV) at the fovea (outer retina and choriocapillaris layers) (C, D). Structural en face OCT (E-H) exhibited a hyporeflective subfoveal lesion with irregular rim and surrounding heterogeneity corresponding to a bacillary layer detachment (BALAD).

A 79-year-old woman without any systemic comorbidities presented for evaluation of left eye vision loss. Initially, BCVA was 20/25 in the right eye and 20/400 in the left, while IOP was 16 mmHg bilaterally. She was pseudophakic in both eyes (left eye cataract was removed a few months ago) with an otherwise unremarkable anterior segment examination. Dilated fundus examination showed early non-exudative AMD (drusen) in the right eye and active neovascular AMD in the left. A round, yellow-gray elevated lesion (CRT = 598 μm) surrounded by a subtle hypopigmented ring, along with minor hemorrhage and exudation, was noticed on the left macula (Figure 5). Spectral-domain OCT revealed the existence of a foveal detachment with photoreceptor splitting at the inner segment myoid, forming a dome-shaped intraretinal cavity with smooth, sharply demarcated walls, located between an intact ELM and the EZ (BALAD) (Figure 6). Besides a shallow fibrovascular PED and subretinal fibrosis, intraretinal fluid (IRF) and subretinal fluid (SRF) can be documented along with the BALAD. OCTA revealed a well-defined, high-flow lacy-wheel/sea-fan neovascular complex located at the outer retina and the choriocapillaris layers, composed of a dense central core with arborizing peripheral fronds, closed loops, and multiple anastomoses (type 2 CNV). Structural en face OCT exhibited coalescent intraretinal cysts in the inner and outer retinal layers and a hyporeflective subfoveal lesion with an irregular rim and surrounding heterogeneity corresponding to BALAD (Figure 7). These OCT findings demonstrate active nAMD with evolution to a BALAD, indicating acute outer retinal decompensation in the setting of high exudative drive from the CNV.

**Figure 8:**
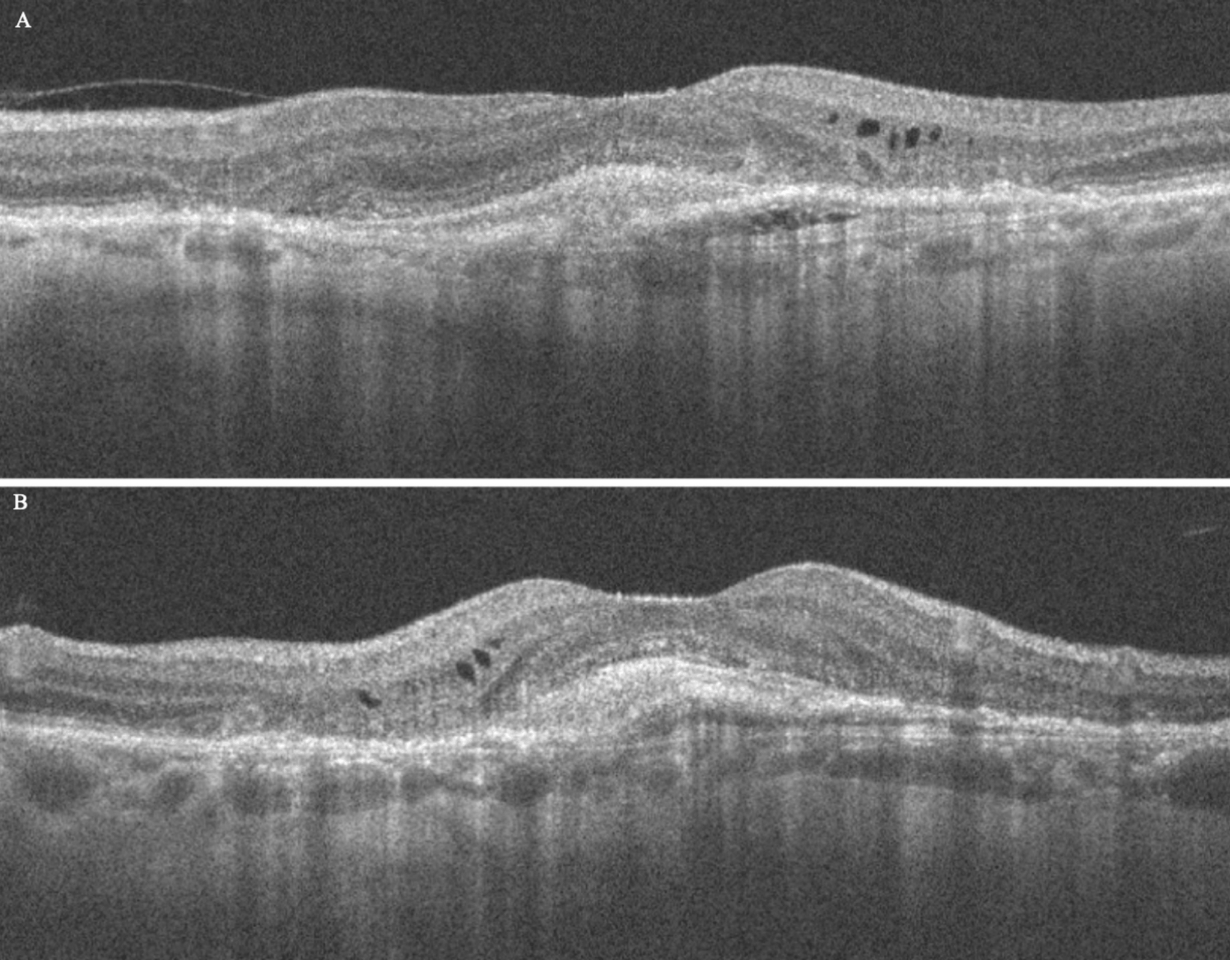
Spectral-domain optical coherence tomography (OCT) of the left eye at presentation (A horizontal scan and B vertical scan), presenting complete resolution of the bacillary layer detachment (BALAD) cavity, resolution of subretinal fluid with a few intraretinal cysts and a flattened scar.

The patient received seven serial intravitreal anti-VEGF injections (aflibercept 2 mg) in the left eye, on a treat-and-extend regimen. At the 10-month follow-up, BCVA OS improved to 20/40, and serial OCT showed complete resolution of the BALAD cavity (CRT = 244 μm), leaving a flattened scar and resolution of SRF (Figure 8). Hence, BCVA gains are correlated with anatomical improvement. Unfortunately, follow-up was then discontinued as the patient was diagnosed with metastatic cancer.

Case 3

**Figure 9:**
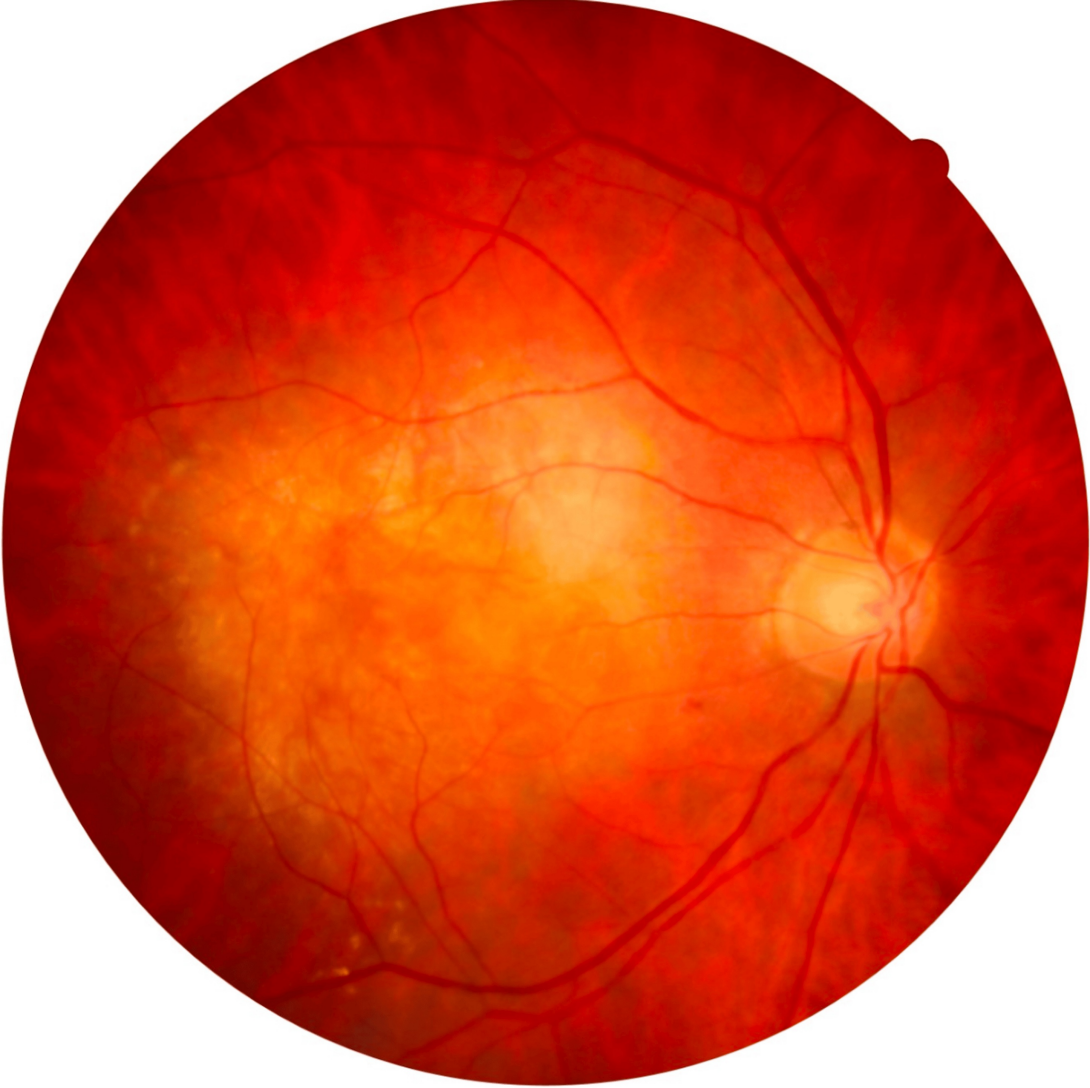
Color fundus photography of the right eye at presentation. A broad, ill-defined yellow-white subfoveal plaque with a fibrotic sheen can be noticed.

**Figure 10:**
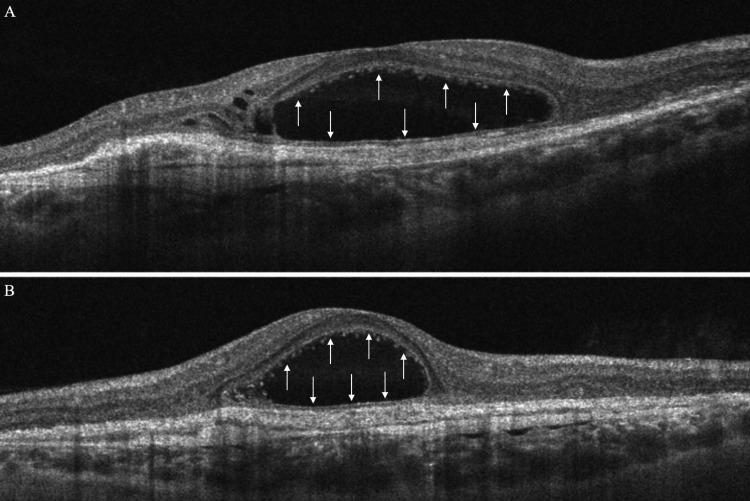
Spectral-domain optical coherence tomography (OCT) at presentation (A horizontal scan and B vertical scan), demonstrating a broad, ellipsoid intraretinal cavity centered at the fovea with smooth, sharply demarcated walls between the external limiting membrane (ELM) and ellipsoid zone (EZ), consistent with a bacillary layer detachment (BALAD) (arrows). Scattered punctate hyperreflective foci line the inner surface of the cavity and outer nuclear layer, possibly fibrinous debris. Small parafoveal cysts are adjacent to the lesion.

**Figure 11:**
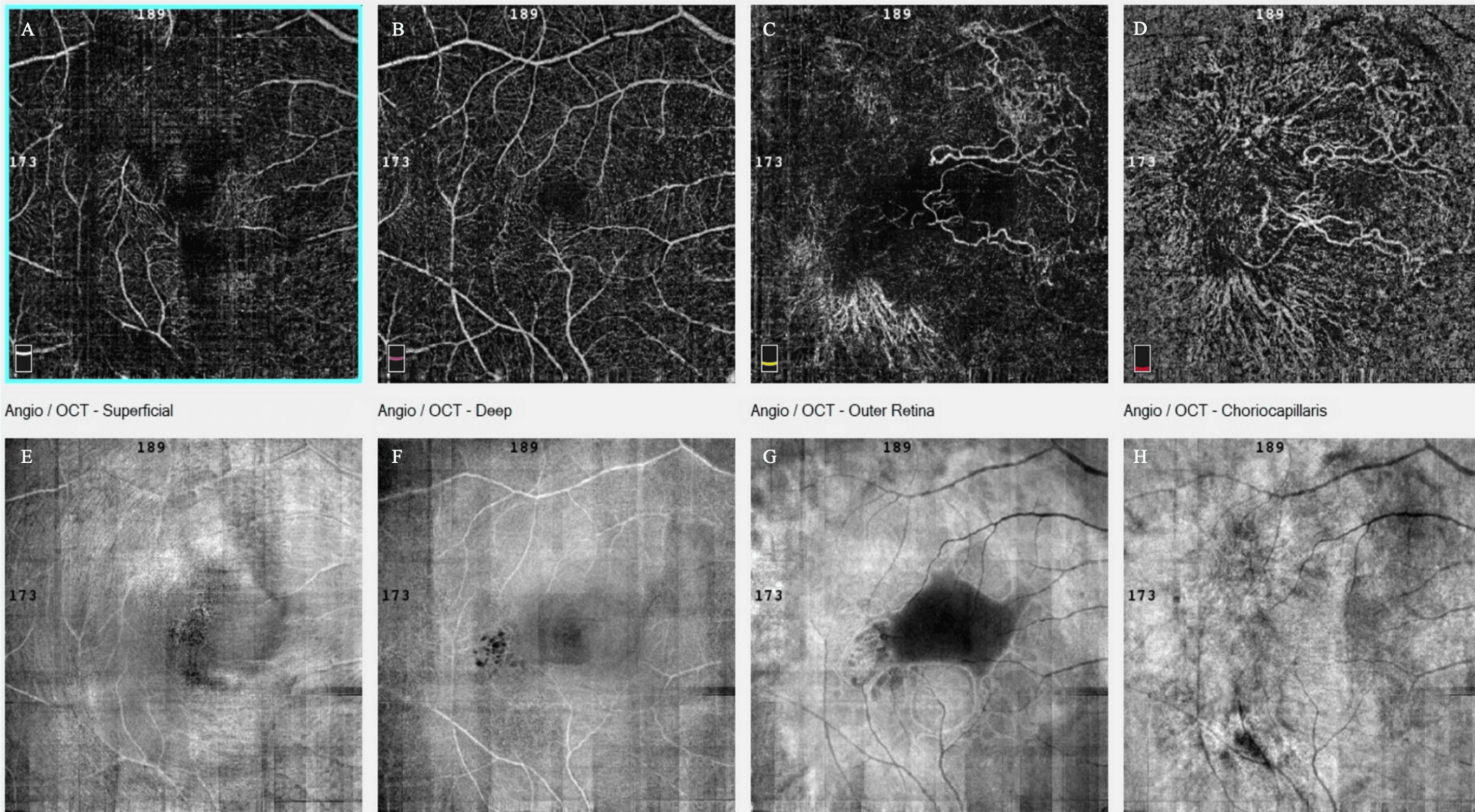
Optical coherence tomography angiography (OCTA) of the right eye (A-D) at presentation, exhibiting an extensive high-flow neovascular complex at the outer retina and choriocapillaris layers. Structural en face OCT (E-H) presented a hyporeflective zone corresponding to bacillary layer detachment (BALAD) with hyperreflective margins (Figure 9).

A 75-year-old man with type 2 diabetes mellitus, dyslipidemia, and hypertension presented with chronic vision reduction in his right eye. Initial BCVA was 20/100 and 20/40 in the right and left eye, respectively, while IOP was 15 mmHg bilaterally. The right eye was pseudophakic, while the left eye had a cataract, and the rest of the anterior segment examination was unremarkable. Dilated fundus examination showed no signs of diabetic retinopathy or diabetic macular edema in both eyes, but in the right eye, a broad, ill-defined yellow-white subfoveal atrophic plaque with a fibrotic sheen was observed (Figure 9). Spectral-domain OCT demonstrated a broad, ellipsoid intraretinal cavity centered at the fovea of the right eye with smooth, sharply demarcated walls between ELM and EZ, consistent with a BALAD. Scattered punctate hyperreflective foci line the inner surface of the cavity and adjacent outer nuclear layer, compatible with fibrinous debris. Small parafoveal cysts are present nasal to the lesion, while there is significant foveal involvement with subretinal fibrosis (Figure 10). OCTA identified a widespread high-flow neovascular complex at the outer retina and choriocapillaris layers with a compact central core and arborizing peripheral fronds forming anastomoses, consistent with type 2 CNV. Structural en face OCT demonstrates exquisitely a central oval hyporeflective area corresponding to the detached layers of the retina, surrounded by a hyperreflective zone at the rims of the lesion (Figure 11). These OCT findings reveal outer-retinal splitting at the photoreceptor inner-segment myoid (BALAD) with mild intralesional reflectivity (fibrinous or blood byproducts). No intravitreal therapy was administered due to the patient’s lack of consent. We were willing to treat him either privately or in the public hospital, but he declined, and the patient was lost to follow-up.

 Table 1 shows a summary of the demographic and clinical characteristics of the three patients with BALAD.

**Table 1:** Demographic and clinical characteristics of patients with BALAD BALAD: bacillary layer detachment, CRT: central retinal thickness, VEGF: vascular endothelial growth factor, BCVA: best-corrected visual acuity, nAMD: neovascular age-related macular degeneration, OS: oculus sinister, OD: oculus dexter

	Gender	Age	Primary disease	Anterior segment	Eye with BALAD	CRT (μm)	Anti-VEGF treatment	BCVA before treatment	BCVA after treatment	Outcome
Case 1	Female	84	nAMD	Cataract	OS	929	Aflibercept 2mg	CF at 1m	20/50	BALAD resolution BCVA improvement
Case 2	Female	79	nAMD	Pseudophakia	OS	598	Aflibercept 2mg	20/400	20/40	BALAD resolution BCVA improvement
Case 3	Male	75	nAMD	Pseudophakia	OD	543	-	20/100	-	-

## Discussion

Our case series attempted to shed more light on a newly described OCT entity in patients with nAMD. The hallmark OCT appearance of BALAD is a dome-shaped, intraretinal cystic space bounded anteriorly by the ELM and posteriorly by the EZ. BALAD often contains granular hyperreflective material (fibrin, photoreceptor debris), which makes its cavity more reflective than typical serous fluid. In some cases, hemorrhage may be present within the BALAD as a bright reflectance material with a shadowing effect. These detachments correspond to splitting at the photoreceptor inner segment myoid and were often adjacent to confluent SRF and hemorrhage [8]. Ramtohul et al. reported BALAD in 14 eyes with type 2 macular neovascularization, all of which featured subretinal hemorrhage and concomitant outer-retinal cystoid spaces [9]. In our cases, multimodal imaging confirmed type 2 CNV underlying each BALAD, mirroring prior observations that BALAD frequently accompanies aggressive CNV phenotypes. Pathophysiologically, BALAD is considered a photoreceptor layer schisis, as outward hydrostatic forces from exudation overcome the relatively weak resistance of the myoid zone of the inner segments, creating an intraretinal cystic split [10].

Published series suggest BALAD is rare in nAMD. In a large retrospective cohort of 442 nAMD patients, the incidence of BALAD was only 4.5% (20/442) [11]. Similarly, a post-hoc analysis of the HAWK trial identified classic BALAD in 7.2% of eyes at baseline. BALAD is found predominantly in type 2 lesions (12.5%) than in type 1 (2.7%) [12]. By contrast, another study reported BALAD across all nAMD subtypes, with type 1 CNV actually predominating (63%). These discrepancies likely reflect sample and referral biases, but they highlight that BALAD can occur in any CNV type. Overall, however, type 2 lesions and hemorrhagic or exudative cases appear to be most prone to BALAD formation [13]. Our cases fit the aforementioned pattern; each case involved a CNV with substantial subretinal exudation.

BALAD is not unique to AMD. It has been reported in numerous disorders, especially those with sudden exudation or inflammation (Table 2). The chief examples are Vogt-Koyanagi-Harada disease (seen in 47% of eyes in some series) and acute posterior multifocal placoid pigment epitheliopathy (APMPPE). Infectious uveitides (e.g., toxoplasmosis retinitis) and pachychoroid disorders (central serous chorioretinopathy) also show BALAD in a minority of cases. Neoplastic conditions (carcinoma-associated retinopathy, choroidal metastasis, melanoma) and trauma have likewise been associated with bacillary splits. In these diseases, BALAD often correlates with active exudation or inflammation and typically resolves as the underlying pathology is controlled [14].

**Table 2:** BALAD-associated diseases AMD: age-related macular degeneration, CNV: choroidal neovascularization, RD: retinal detachment, APMPPE: acute posterior multifocal placoid pigment epitheliopathy, BALAD: bacillary layer detachment, SRF: subretinal fluid, IRF: intraretinal fluid

Category	Condition / examples	Typical clinical context
Neovascular maculopathies	Neovascular AMD (type 1 and type 2 CNV), polypoidal choroidal vasculopathy	Elderly; high exudation ± subretinal hemorrhage
	Myopic CNV, angioid streaks–related CNV, inflammatory CNV	Pathologic myopia, pseudoxanthoma elasticum, uveitis history
Pachychoroid spectrum	Central serous chorioretinopathy (acute), pachychoroid neovasculopathy	Middle-aged; stress/steroids; thick choroid
Inflammatory uveitides	Vogt–Koyanagi–Harada, sympathetic ophthalmia	Bilateral serous RD; meningeal/auditory symptoms
	White-dot syndromes (e.g., APMPPE, multifocal choroiditis)	Outer-retinal disruption; occasional BALAD with SRF
Infectious posterior uveitis	Toxoplasmic retinitis, focal retinochoroiditis	BALAD adjacent to active focus; hyperreflective inflammatory material
Retinal vascular disease	Retinal vein occlusion	Acute macular edema
	Diabetic macular edema	Diffuse IRF; occasional bacillary split in severe edema
Retinal Detachment, Effusion disorders	Exudative RD (posterior scleritis, uveal effusion, choroidal hemangioma)	Pain (scleritis); thickened choroid
	Acute rhegmatogenous macula-off RD	High-dome SRF; BALAD at the fovea in some cases
Trauma	Blunt ocular trauma / commotio retinae	Recent impact
Intraocular tumors	Choroidal melanoma, metastasis, circumscribed hemangioma	Mass on exam/imaging

The principal differential diagnoses for BALAD on OCT include outer retinoschisis, true subretinal fluid, and hemorrhage. Outer retinoschisis in the macula (e.g., juvenile X-linked retinoschisis or degenerative lamellar schisis) typically involves splitting at the outer plexiform layer or Henle fiber layer, anterior to the photoreceptor bands, and is usually not associated with a large cystic cavity at the fovea. Unlike BALAD, true subretinal fluid lies between photoreceptors and the RPE, with the entire photoreceptor layer (including the bacillary layer) displaced as a unit. By contrast, BALAD fluid is intraretinal (within the photoreceptor layer), and the EZ often remains intact at its posterior margin. Subretinal fluid is generally optically clear, whereas BALAD cavities frequently contain more hyperreflective material. On fluorescein angiography, BALAD can show pooling but typically no active leakage, distinguishing it from cystoid macular edema or schitic cavities. Also, hemorrhage within or below the retina can mimic BALAD. However, acute submacular hemorrhage usually casts a shadow and obliterates layering, whereas a hemorrhagic BALAD shows blood-level hyperreflectivity within the bacillary cavity. Importantly, recognizing BALAD avoids misdiagnosis of a “giant retinal cyst” or neoplasm [15].

Treatment responses in our series were consistent with the literature. In the first two cases, the BALAD promptly collapsed after intravitreal therapy with aflibercept. The third patient declined treatment. Jung et al. reported complete resolution of BALAD in every eye after intravitreal anti-VEGF therapy, with mean BCVA improving from 20/140 to 20/60 [16]. Likewise, another study found that every BALAD in their series resolved following anti-VEGF therapy by 48 weeks with either brolucizumab or aflibercept [17]. We also noted corresponding improvements in macular anatomy (decreased SRF and CRT) on serial OCT, while visual gains were notable with BALAD resolution. Importantly, eyes with BALAD tend not to achieve the same level of ultimate BCVA as nAMD eyes without BALAD [18].

In nAMD, the presence of BALAD appears to mark a more aggressive disease course and guarded prognosis. Long-term follow-up of BALAD patients shows a high risk of scarring, as it has been noted that 77% of eyes with BALAD developed subretinal fibrosis by 4 years, and mean BCVA regressed toward baseline despite initial improvement [19]. Similarly, it has been emphasized that BALAD is a marker of high-exudation volatility and predicted lower long-term BCVA. In our case series, two of the three patients responded well to the initial intravitreal anti-VEGF therapy with BCVA enhancement and BALAD resolution. Unfortunately, during the first year of follow-up, both patients perished due to heart failure and gastric cancer, so long-term data are not available. Evidence suggests that BALAD is a particularly aggressive nAMD subset; although intravitreal anti-VEGF therapy can lead to resolution of the intraretinal fluid, photoreceptor damage and fibrous metaplasia are often irreversible [20].

This study’s strengths include the detailed multimodal imaging of a rare OCT biomarker in patients with nAMD. By correlating clinical and imaging data, we add evidence on BALAD’s features and outcomes. However, the series is limited by its small size (n = 3), retrospective design, and lack of a control group, precluding incidence estimates or formal statistical analysis. Selection bias is inherent, as we present unusual cases. These limitations underscore the need for larger, prospective studies.

## Conclusions

BALAD is an uncommon OCT biomarker in neovascular AMD that signifies a highly exudative, often hemorrhagic form of CNV. In our series, as in prior reports, BALAD appeared as an outer-retinal split in association with type 2 CNV and resolved rapidly with anti-VEGF treatment. However, these eyes remain at high risk for subretinal fibrosis and limited visual recovery. Clinicians should be vigilant for BALAD on OCT imaging of nAMD patients, as its recognition has prognostic and management implications. Early, aggressive anti-VEGF therapy is indicated, and patients should be counseled about the potential for scar formation. Further research is needed to clarify the pathogenetic mechanisms and the proper management of BALAD in nAMD.
